# Evaluation of Wireless Phosphine Sensors for Monitoring Fumigation Gas in Wheat Stored in Farm Bins

**DOI:** 10.3390/insects10050121

**Published:** 2019-04-27

**Authors:** Daniel Brabec, James Campbell, Frank Arthur, Mark Casada, Dennis Tilley, Sotiris Bantas

**Affiliations:** 1 USDA, Agricultural Research Service, Center for Grain and Animal Health Research, 1515 College Avenue, Manhattan, KS 66505, USA; james.campbell@ars.usda.gov (J.C.); frank.arthur@ars.usda.gov (F.A.); mark.casada@ars.usda.gov (M.C.); dennis.tilley@ars.usda.gov (D.T.); 2 Centaur Analytics, Inc., 1923 Eastman Avenue, Suite 200, Ventura, CA 93003, USA; sotiris@centaur.ag

**Keywords:** fumigation, wireless, grain, instrumentation

## Abstract

Fumigation of grain bins with phosphine tablets is one method of insect control for stored products. Monitoring the concentration of the toxic gas at many locations over several days for a given structure or container can be challenging. In this study, a commercially-available system that wirelessly measures phosphine was evaluated in small-scale and large-scale tests. Small-scale testing was performed to study the repeatability and accuracy of the sensors. The wireless sensors were within 30 ppm of each other, over a range of 700 ppm phosphine. Large-scale testing evaluated the system during the fumigation of wheat stored in 7 m diameter, 120 metric ton, steel grain bins. As a reference, monitoring lines were distributed at several positions and depths in the bin in order to sample phosphine gas concentrations. A series of three fumigation trials were performed, with each lasting for over six days. The wireless devices collected local phosphine concentrations and temperatures every two hours without assistance from personnel. Although the fumigation trials were significantly different in terms of patterns in gas concentration over time, the two sampling methods gave similar trendlines. However, the automated data provided a more detailed picture of the fumigation process. This information may help fumigation managers to better evaluate fumigations and assure successful insect control.

## 1. Introduction

The fumigation of grain bins with phosphine is a commonly used method of controlling insect infestations in stored grains [1,2]. While the monitoring of fumigant concentration over time is critical for determining the effectiveness of fumigations, actual measurements are often not taken or are limited in scope. This can lead to fumigation failures, due to not obtaining target concentration/exposure time levels in all, or in parts, of the bin. Improper fumigations have led to strains of pests that are strongly resistant to normal fumigation methods involving phosphine [3,4]. Lack of monitoring is due to a range of factors. Grain bins can be difficult to access, and often require the use of climbing ladders on their sides to reach the top of the bins. Entering the bin headspace requires special safety considerations according to the US Occupational Safety and Health Administration, OSHA, Grain Handling Facilities Standard [5]. Many commercial operations limit bin access due to these safety concerns. In addition, it can be difficult to get data on phosphine concentration from different locations within the grain mass, as it requires advance planning and considerable labor and costs to obtain data from deep in the grain mass. These factors contribute to the difficulties involved in monitoring fumigation and validating phosphine production and containment.

Typical approaches for monitoring phosphine gas concentration in grain bins involve the introduction of monitoring lines or tubing into different depths of the grain or the headspace, and then drawing air samples periodically through these tubes and measuring their phosphine concentration. Sampling airlines can be permanently or temporarily installed in fumigated spaces, and extracted gas samples can be measured with gas meters or Dräger tubes. Manually monitoring gas concentrations in this way is time consuming, and sampling at multiple locations is limited due to the difficulty involved in the introduction of lines into the grain and the need for people to be available at regular time intervals, especially during the night, to take the samples. Previous research describes a monitoring system that used 23 permanently installed sampling airlines in two metal grain bins (each ~50 metric tons) [6]. This system was effective for monitoring phosphine concentrations during four fumigations of each bin, and it was able to indicate spatial variation in gas concentration, including identifying areas that were not achieving target concentration/exposure times. However, the system was also labor intensive, and the monitoring frequency was dependent on worker availability. A more automated method of monitoring the fumigations would reduce labor requirements and lead to improved fumigations. 

Wireless sensors and cloud-based systems are increasingly used in agricultural systems. Five areas in the agriculture and food industries were identified where wireless sensing systems are being developed; environmental monitoring, precision agriculture, machine and process control, facility automation, and traceability systems [7]. Wireless sensing has also been applied to IR temperature monitoring of crops and to controlling center pivot irrigations [8,9,10]. A system was developed for remotely monitoring the carbon dioxide levels of the headspace and the fan exhaust of commercial corn storages to detect elevated CO_2_, as produced from grain molding. The CO_2_ sensors communicated wirelessly via one radio to a second radio, and then to a computer [11]. The use of wireless technology for monitoring fumigations would, in addition to being less labor intensive, be an inherently safer technique because of the fact that the monitoring information is acquired at further distances from the grain bins or treatment sites, and would consequently avoid the potential toxic off-gas within the proximity of those structures. Early remote monitoring of phosphine was performed inside and outside of five tobacco warehouses [12]. Their system included radio telemetry and used sensors to successfully monitor the phosphine concentration inside the tobacco warehouses over 12 days. This test also included extensive monitoring around the border of the property for phosphine level ~0.3 ppm for personnel safety. 

Recently, a commercially available system that includes phosphine sensors, wireless communications, data collection software, data analysis, and internet access has been developed [13]. The use of wireless sensors and supporting electronics face additional challenges with phosphine fumigations, because phosphine is corrosive to many metals, such as copper. This commercial system and its packaging include features to protect these electronics from exposure to phosphine. The system has been previously tested with fumigations of shipping containers, warehouses, and ship-holds, but not in bulk storage. The wireless sensors proved valuable for monitoring fumigation, and demonstrated that fumigation in air-tight shipping containers was able to kill the lesser grain borer and the saw-toothed rice beetle using bioassays. The monitoring of fumigation in the warehouse and ship-hold showed reduced concentrations and holding times, and reduced insect efficacies relative to the shipping container [14]. The objective of this study was to evaluate the accuracy and responsiveness of the system when placed in the bulk grain stored in grain bins. Several fumigations of wheat stored in 120 metric ton steel grain bins were performed, and gas concentration was measured. The wireless system was compared with other results that used traditional hand-held meter and airlines.

## 2. Materials and Methods 

### 2.1. Wireless Phosphine System

**Figure 1:**
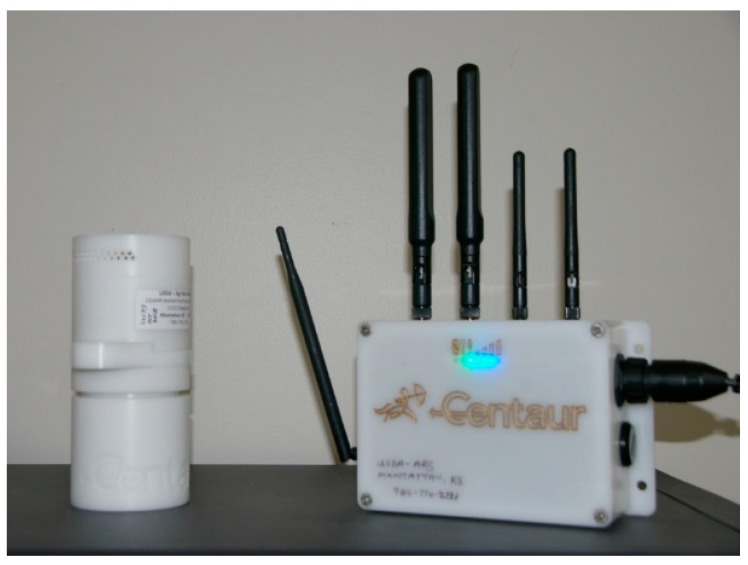
Photograph of the wireless sensor on the left and communication electronics on the right.

The basic components of the system (Centaur Analytics, Ventura, CA, USA) included a plastic housing (8 cm diameter × 17 cm long) holding phosphine and temperature sensors, sleep/wake timing electronics for battery power conservation, and wireless interface communications. Additional electronics were mounted outside of each bin to receive signals from the sensors, temporarily store data, and to send data to the cellular-interface electronics (Figure 1). The cell phone connection transferred the data to cloud-based software, which allowed easy access to the fumigation data from remote locations. Initial setup of the wireless phosphine sensors was done through the web-based software, which included identifying each sensor, and logging its location and the data collection frequency. 

### 2.2. Sensor Repeatability Testing

**Figure 2:**
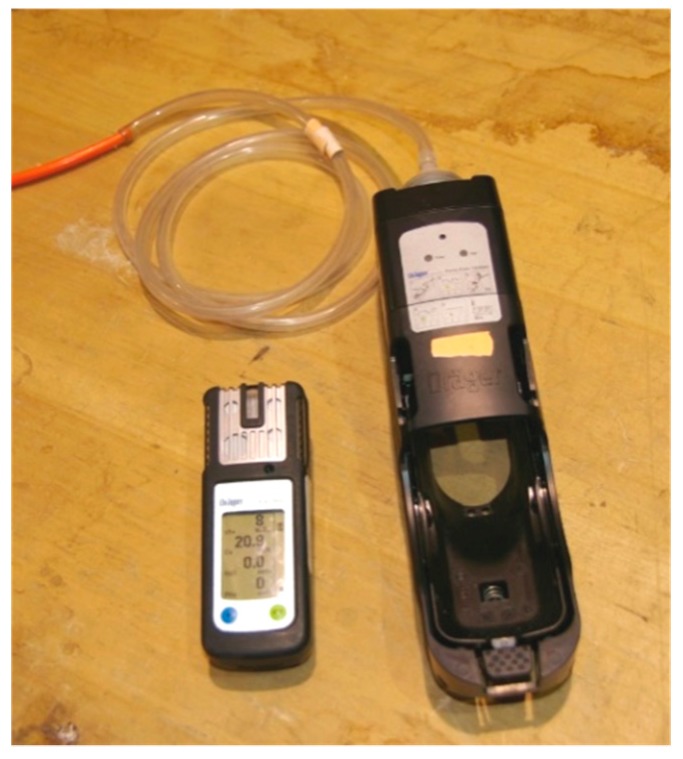
Photograph of Dräger X-am 5000 hand-held meter and air sample tubing.

A small-scale test was used to evaluate the repeatability and accuracy of the four wireless sensors, which were positioned closely together inside a barrel. A 208 L barrel was loaded half full with ~70 kg of hard-red winter wheat. The grain was ~12% moisture content and the barrels were maintained at ~25 °C. A plastic monitoring line (6.4 mm diameter and ~5 m long) was placed in the middle of the wheat and routed through the lid of barrel to enable the monitoring of the phosphine levels with a hand-held meter (X-am 5000, Dräger, Lubeck, Germany). Air was drawn through the tubing to the hand-held meter (Figure 2) using an air-pump (model 1/2/5000, Dräger, Lubeck, Germany).

Four of the wireless phosphine sensors were placed on the wheat and were equally spaced around the center of barrel. A single Phostoxin pellet (Degesch America, Weyers Cave, VA, USA) was placed into the wheat. A single Phostoxin pellet contains 0.6 g aluminum phosphide and produces ~0.2 g phosphine_._ The lid of the barrel was sealed and the barrel was monitored over two days using both the hand-held meter and the wireless sensors. Manual measurements through the monitoring lines with the hand-held meter were recorded every 2 h between 8:00 and 20:00, while the wireless sensors recorded phosphine concentrations every hour. 

The phosphine concentration that was recorded by the four sensors was averaged and then compared to the standard deviation as an indication of repeatability. The average phosphine measurements from the wireless sensors were also compared to the hand-held meter readings. The difference between the two measurements was divided by the concentration measured by the hand-held meter to determine the percentage difference across the range of concentrations tested. 

### 2.3. Fumigation Monitoring Experiments

**Figure 3:**
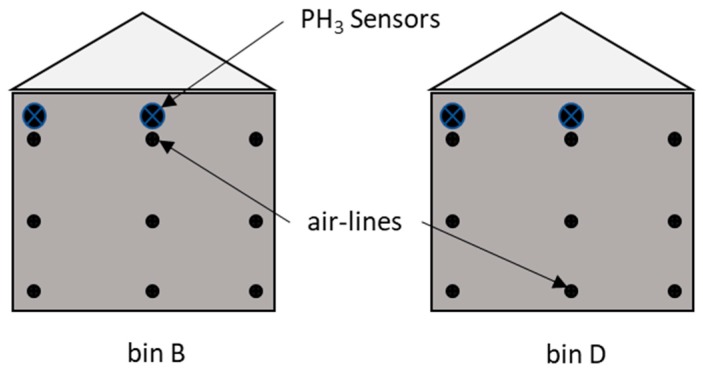
Schematic side-view of two grain bins and the locations of the four wireless phosphine sensors compared to the air sampling locations.

The primary studies involved the monitoring of several fumigation trials. Each trial used two steel grain bins, which were located at the USDA-ARS facility in Manhattan, Kansas. Each bin was 6.7 m in diameter with 4.3 m side walls. The bins were filled to a depth of ~3.7 m with ~110 metric tons of wheat. The air temperature ranged from 27 °C down to 18 °C, with humidity averaging 45%. The grain moisture content was ~11.5%. The grain bins were sealed at the roof vents, the roof eaves, and at the aeration fans. Plastic monitoring lines (6.4 mm diameter) were distributed within the grain bin, with the ends of the monitoring lines located at depths of 0.1 m (top), 1.8 m (middle), and 3.3 m (bottom). Two wireless phosphine sensors were placed in each bin, with one at the bin center and the other ~0.3 m away from the south wall (Figure 3). The sensors were placed ~10 cm under the grain surface and within 10 cm of the end of one of the monitoring lines. Because of the limited number of wireless sensors available, and the attempt to duplicate measures, sensors were placed on the top of the grain, possibly as a farmer or user might do.

A series of three fumigation trials were completed. Degesch Phostoxin tablets (Degesch America, Weyers Cave, VA, USA) were used for the fumigations. A single Phostoxin tablet contains 3.1 g aluminum phosphide and produces ~1.0 g phosphine_._ The application manual that was provided with the phosphine tables recommended a 2.5 to 5.0 g/m^3^ dosage when applied to farm bins, depending on grain temperature. For the grain volume in these tests, 320 tablets and 640 tablets were used, which provided dosages of 2.3 and 4.6 g/m^3^ of phosphine. For fumigation trial #1, 320 tablets were probed into the top 1 meter of the grain at nine locations, including the center and two locations along each north, south, east, and west axis. For fumigation trials #2 and #3, the tablets were not probed, but rather distributed over the top of the grain. For trial #2, 320 tablets were used, and for trial #3, 640 tablets were used. The dosages in each trial were sub-divided into containers holding 20 tablets each. The closed containers of chemicals were placed into buckets and carried up the ladder and into the top of the wheat. Then, the containers were equally distributed onto the top of the grain and emptied evenly on the top surface of the grain. For trials #2 and #3, a recirculation fan was operated intermittently, every six hours for 45 minutes, to help distribute the phosphine gas in the wheat.

The gas concentrations during fumigation were monitored for ~7 days using both the wireless system and manually using the monitoring lines and hand-held meter. The wireless system was programed to collect data on phosphine concentration and temperature every 2 h while the manual sampling was conducted 1–3 times per day. After 36 h of fumigation, the readings of the wireless sensor located in the center of bin B were compared to the readings of the monitoring lines and hand-held meter for each fumigation trial.

## 3. Results and Discussions

### 3.1. Sensors Test Results

**Figure 4:**
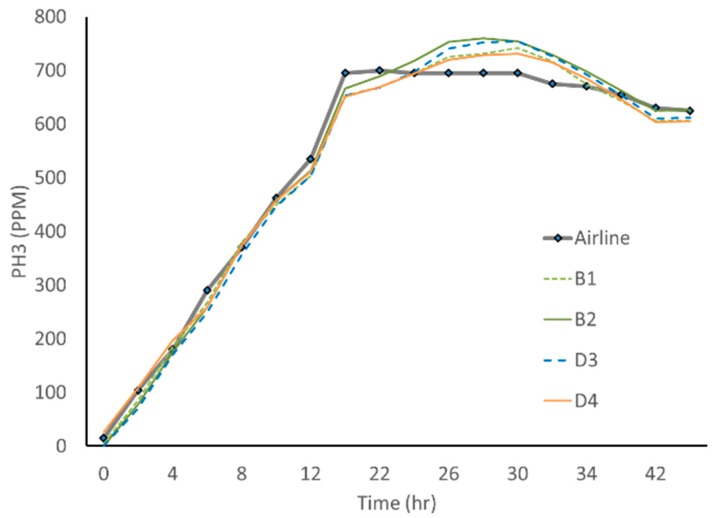
The repeatability and relative accuracy chart for four phosphine sensors compared to the hand-held meter. Sensors B1, B2, D3, and D4 were placed into a barrel together along with an airline. B1 and B2 were wireless sensors used later in bin B, while D3 and D4 were wireless sensors used in bin D.

The preliminary test measured variation among the four sensors (Figure 4). One phosphine sensor appeared to have an ~25 ppm offset from 0 ppm which caused a higher error at gas concentrations below 100 ppm. The standard deviation of the four sensors ranged from 5 to 15 ppm per observation, or 1% to 15% for individual measured concentrations. The standard deviation averaged 10 ppm for all measurements ranging up to 700 ppm. If the gas concentration was ~300 ppm, then an individual sensor should read within ±20 ppm, or ~ 3% for 95% of the observations, or two standard deviations.

To determine relative accuracy, the average wireless sensor values were compared to our reference measurements from the hand-held meter. Although this meter is not an analytical method such as gas chromatography, it is a commonly used method for measuring gas concentrations, so we used it as the standard. This hand-held meter has a range of 0–2000 ppm of phosphine, a resolution of 1 ppm, and is commonly accurate to 2% of full scale. When the phosphine gas was generated and the concentration was changing, the wireless sensors closely matched the hand-held meter, as seen in Figure 4. At the peak concentration, the hand-held meter recorded level concentrations while the wireless sensor concentration values drifted higher. It is during this period of relatively constant phosphine that the wireless sensors differed the most among themselves, and the airline readings and the concentration measurements varied by around 7%. The exact reason for this difference is not certain. However, both measurements are showing adequate phosphine concentrations for lethal fumigation. Current USA grain fumigation practices for grain bins recommended holding 200–300 ppm of phosphine concentration for 3–4 days, depending on temperature, to kill all insect life stages [15]. Although these systems are not highly precise, the wireless sensor can monitor the gas concentration and time adequately and safely in order to determine the total concentration x time, which can be compared with store product insect efficacies.

There are several devices available for measuring phosphine concentrations. For stored grain fumigations, the phosphine concentrations usually range from 100–1000 ppm. Dräger gas sampling tubes are available and made for this range and have scaled marking for every 100 ppm. Thus, the accuracy of these tubes is approximately half of the scale, or ~50 ppm. And the use of Dräger tubes to monitor fumigations requires additional respiratory safety equipment, because it places the applicator in close proximity to the fumigation and potential toxic gas. Many other instrument manufacturers that have devices for sampling phosphine with monitoring lines place the applicator at safer distances. These companies include LumaSense Technologies (Santa Clara, CA, USA), Bedfont Scientific (Lahore, Pakistan), Gastech (Wangara, Western Australia, AU), and Spectros Instruments (Hopedale, MA, USA). Some of these instruments use solid-state electronic sensors with ~2% accuracy of full scale, while others use infrared sensor technology which has better than 1% accuracy [16]. For worker safety, personal monitors are used, however they are designed to measure and alarm at the level of ~3 ppm of phosphine [1,17,18].

### 3.2. Fumigation Test Results 

**Figure 5:**
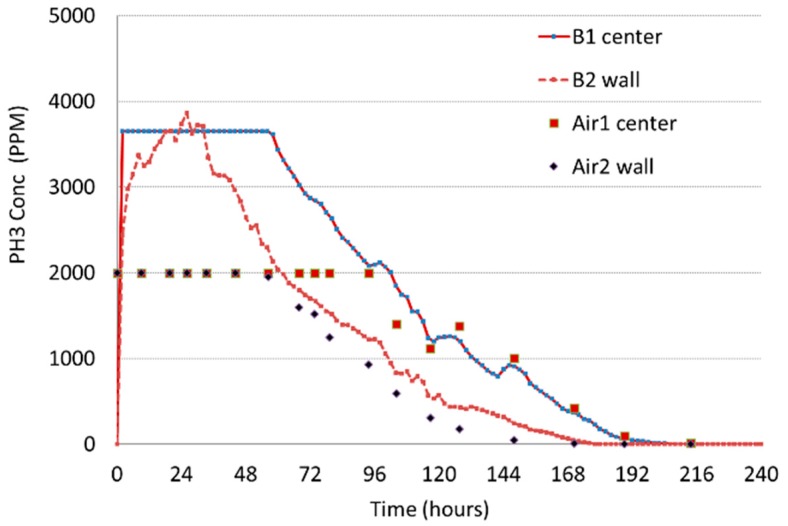
Fumigation Trial #1: 320 Phostoxin tablets were probed into the top of grain and had no supplemental airflow. B1 and B2 are the wireless sensors in bin B while Air1 and Air2 are the airlines positioned next to the sensors.

Fumigation trial #1 used 320 tablets that were probed into the grain and no additional airflow for circulation. The two measurement methods showed very different results in the first 72 h (Figure 5). 

Phosphine increased very rapidly to very high concentrations. The different sensors became saturated, stopped accurately measuring at different points, and remained saturated for different times. The wireless sensors could record higher phosphine concentrations, but interestingly, one wireless sensor at the center location became saturated at 3600 ppm while the sensor at the wall location did not become saturated, and even collected data above 3600 ppm. It could be that the rate of increase in concentration was different between these two locations, as well as there being possible differences in total concentration. The airline measurements became saturated at 2000 ppm. The Dräger X-am 5000 meter has a maximum range of 2000 ppm. As the phosphine gas concentrations were reduced to below 2000 ppm, the wireless sensors and the hand-held meter data followed similar trends. Although the airline readings tended to be slightly lower than the wireless sensor readings at the wall position, the center readings from both methods were similar.

**Figure 6:**
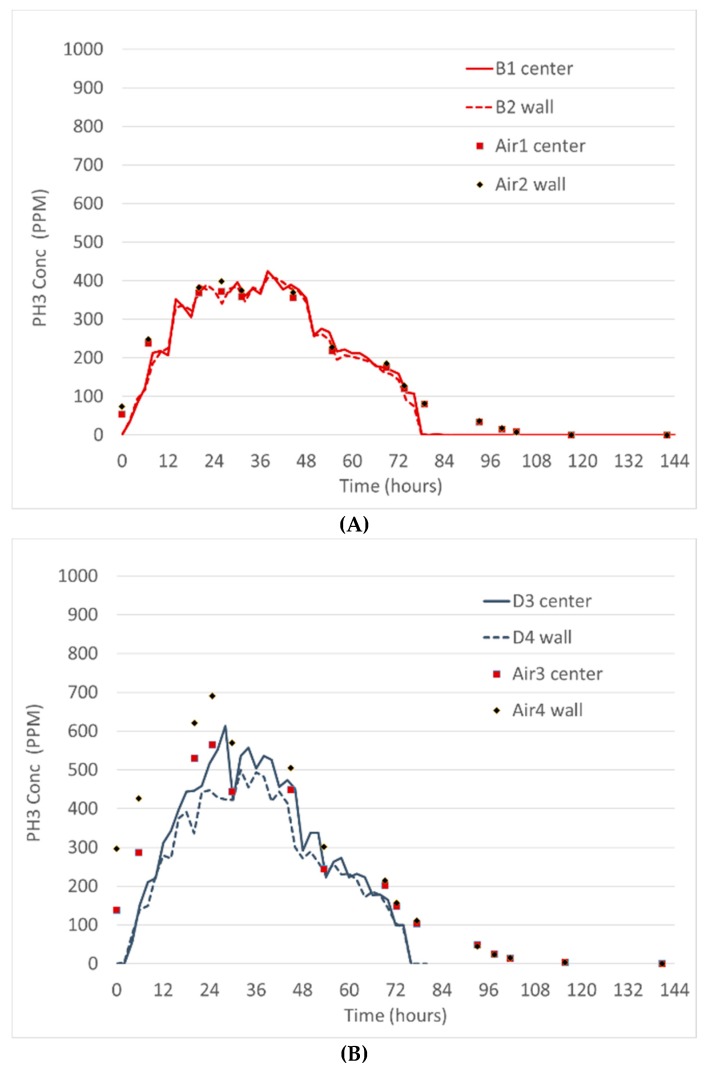
Fumigation Trial #2. (**A**) Bin B with 320 Phostoxin tablets distributed on the grain surface with a recirculating fan providing intermittent airflow. B1 and B2 are the wireless sensors while Air1 and Air2 are the airlines next to those sensors. (**B**) Bin D was prepared, treated, and monitored with the sensors and the airlines at the same time. D3 and D4 are the wireless sensors while Air3 and Air4 are the airlines next to them.

Fumigation trial #2 used 320 tablets, but recirculation fans were periodically operated to distribute the phosphine. Graphs of the fumigation from two different grain bins show similar trends when the gas holding time was over 72 h. Bin B had maximum concentrations of less than 400 ppm while the bin D concentrations were over 500 ppm (Figure 6).

**Figure 7:**
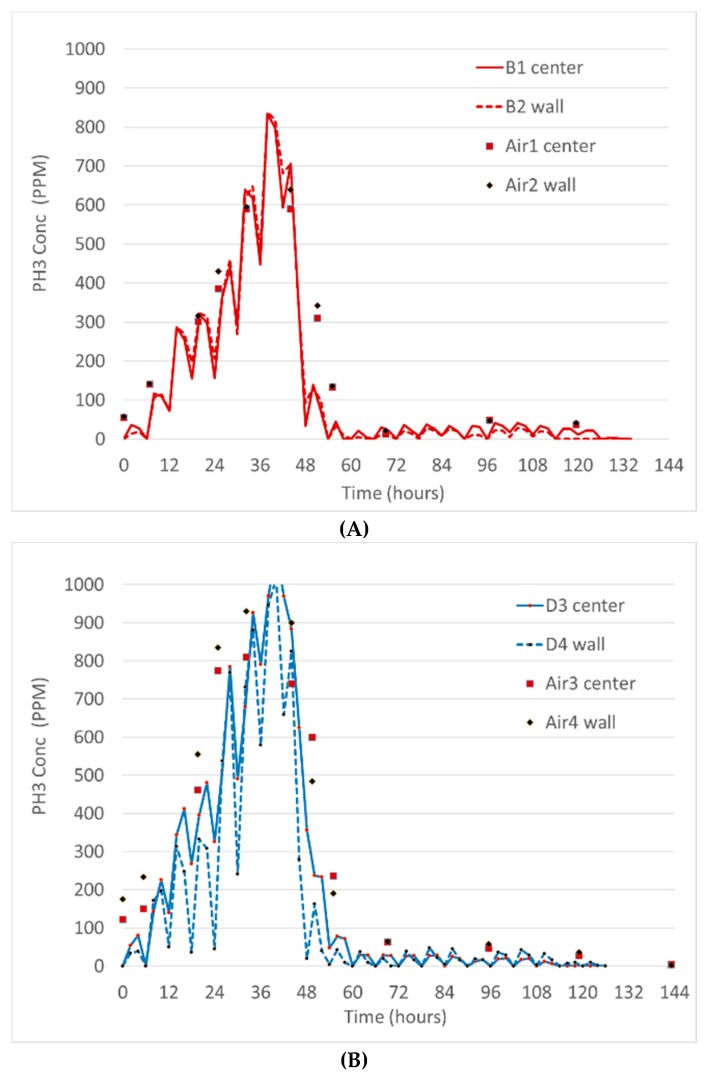
Fumigation Trial #3. (**A**) Bin B with 640 Phostoxin tablets distributed on grain surface with periodic operation of recirculation fan. (**B**) Bin D was prepared, treated and monitored with sensors and airlines at the same time.

Fumigation trial #3 used 640 tablets and intermittent recirculating fans. The gas holding time was less than 55 h. Bin B had maximum concentrations of over 800 ppm, while bin D had concentrations over 1000 ppm. As seen in Figure 7, a severe drop in phosphine concentration was observed ~48 h after the start of the trial, which coincided with a thunderstorm moving through the region. Figure 7 clearly displays sub-cyclic changes in the phosphine concentrations, which coincide with the operation of the recirculation fan. These sub-cycles are evident with the wireless sensors and not the monitoring line data, because data was more frequently and consistently recorded with the wireless sensors.

Phosphine is an important fumigation option that is widely used in the grain industry. However, poor fumigation practices have led to inadequate concentrations and holding times for the control of insect infestations [19,20]. This allows the development of phosphine-resistant populations, which could be even more difficult to control in future fumigations [21]. It is important to use optimal fumigation methods to mitigate the development of resistance. The wireless phosphine monitoring methods could help validate good fumigation treatments or identify situations where fumigation levels were too low, or when the gas was not held for a sufficient time, to ensure the complete kill of insects.

### 3.3. Phosphine Concentration at Various Grain Bin Depths 

We have focused on comparing the phosphine concentrations between the two sensors, and as such, only some of the data collected using the monitoring lines has been reported. The grid of airlines was much more extensive and this data will be analyzed more fully elsewhere, but here we show data from different depths during the three trials to highlight the variation in phosphine concentrations (Table 1). These additional observations help to show one limitation of these measurements, which is that the concentration can vary considerably with depth in the grain mass and the readings from both methods only provide data for localized conditions. 

Trial #1 did not use fans to move the phosphine. This resulted in concentrations of over 2000 ppm at the top of the grain mass, and apparently the phosphine did not reach deeper locations within the grain. In trials #2 and #3, with recirculating air to move the phosphine throughout the bins, the concentrations showed greater similarity at different depths. In order to get a better representation of phosphine concentration throughout the depth of the grain, either additional sensors are needed to be installed at lower depths, or some type of mathematical modeling could be utilized to estimate these extreme differences in gas movement within the grain mass. 

The non-uniform distribution of phosphine in large grain bins filled with wheat was observed in bins without recirculation fans in Australia. Phosphine blankets were placed in the headspace where high concentrations of phosphine were generated and measured. During the cooler fall season, it took ~48 h, and during the hot summer season, it took ~120 h before convective air currents moved phosphine to the bottom of the bin [22]. Our results show that the phosphine in the bins used in this study was not held sufficiently long enough for these longer-range patterns to materialize.

Recirculating airflow improved the distribution of the phosphine. Closed-loop fumigation system designs for central USA [23] and for Australia [24] are available and list the basic features and operations of good fumigation recirculation systems. A gas monitoring strategy should be included with these systems to monitor the concentrations and holding times. Additionally, gas distribution models can be used with the monitoring hardware to estimate variation in concentrations within the grain mass. 

## 4. Conclusions

The wireless phosphine sensors provided a convenient way to monitor fumigation treatments which involved less labor than the hand-held phosphine meter and the air sampling system. The repeatability of the four wireless sensors in this study was usually within ±20 ppm of each other, and their gas concentration measurements were close to our reference hand-held meter. Data was collected easily and more frequently with the wireless system, which provided more detail of the changes in phosphine concentration during treatments, such as changing weather events or recirculation fan effects. Systems that use an internet interface provided convenient access to the data, both while the fumigation was active and when summarizing the results of treatments. Future tests may include other containers that are difficult to monitor such as fumigated railcar, barges, or ship-holds. 

## Figures and Tables

**Table 1:** Phosphine concentrations (ppm) measured with the wireless sensor at the top and with monitoring lines at varied grain depths in bin B after 36 h of fumigation. Trial #1 used 320 tablets and static air. Trial #2 used 320 tablets and recirculating airflow. Trial #3 used 640 tablets and recirculating airflow.

Grain	Trial #1		Trial #2		Trial #3	
Depth	Sensor	Airline	Sensor	Airline	Sensor	Airline
Top	3600	2000	380	360	600	590
Mid.	-	4	-	325	-	550
Bot.	-	0	-	180	-	490
